# 4-Methyl-2-oxo-2,3-dihydro-1-benzopyran-7-yl benzene­sulfonate

**DOI:** 10.1107/S1600536808032005

**Published:** 2008-10-09

**Authors:** Shu-Ping Yang, Da-Qi Wang, Li-Jun Han, Yu-Fen Liu

**Affiliations:** a School of Chemical Engineering, Huaihai Institute of Technology, Lianyungang 222005, People’s Republic of China; b College of Chemistry and Chemical Engineering, Liaocheng University, Shandong 252059, People’s Republic of China; c School of Mathematics and Science, Huaihai Institute of Technology, Lianyungang 222005, People’s Republic of China

## Abstract

The title compound, C_16_H_12_O_5_S, is a derivative of coumarin. The dihedral angle between the coumarin ring system and the phenyl ring is 65.9 (1)°. In the crystal structure, mol­ecules are linked by weak C—H⋯O hydrogen bonding to form molecular ribbons.

## Related literature

For general background, see: Xie *et al.* (2001 ▶); Tanitame *et al.* (2004 ▶); Shao *et al.* (1997 ▶); Rendenbach-Müller *et al.* (1994 ▶); Pochet *et al.* (1996 ▶); Yang *et al.* (2007 ▶, 2006 ▶). For a related structure, see: Yang *et al.* (2007 ▶).
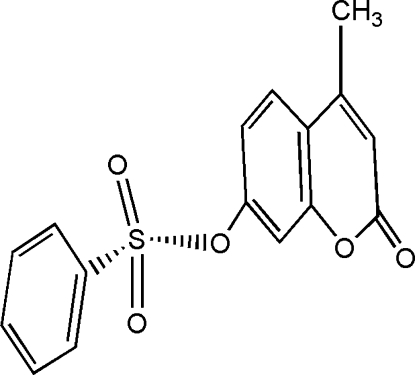


## Experimental

### 

#### Crystal data

C_16_H_12_O_5_S
*M* *_r_* = 316.32
Orthorhombic, 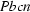

*a* = 23.319 (3) Å
*b* = 9.0865 (12) Å
*c* = 13.7280 (17) Å
*V* = 2908.8 (6) Å^3^
*Z* = 8
Mo *K*α radiation
μ = 0.24 mm^−1^
*T* = 298 (2) K
0.48 × 0.35 × 0.23 mm

#### Data collection

Siemens SMART CCD area-detector diffractometer
Absorption correction: multi-scan (*SADABS*; Sheldrick, 1996 ▶) *T*_min_ = 0.892, *T*_max_ = 0.946
11238 measured reflections
2557 independent reflections
1340 reflections with *I* > 2σ(*I*)
*R*_int_ = 0.077

#### Refinement

*R*[*F* ^2^ > 2σ(*F* ^2^)] = 0.049
*wR*(*F* ^2^) = 0.164
*S* = 1.09
2557 reflections
200 parameters
H-atom parameters constrained
Δρ_max_ = 0.21 e Å^−3^
Δρ_min_ = −0.27 e Å^−3^

### 

Data collection: *SMART* (Siemens, 1996 ▶); cell refinement: *SAINT* (Siemens, 1996 ▶); data reduction: *SAINT*; program(s) used to solve structure: *SHELXTL* (Sheldrick, 2008 ▶); program(s) used to refine structure: *SHELXTL*; molecular graphics: *SHELXTL*; software used to prepare material for publication: *SHELXTL*.

## Supplementary Material

Crystal structure: contains datablocks I, global. DOI: 10.1107/S1600536808032005/xu2456sup1.cif

Structure factors: contains datablocks I. DOI: 10.1107/S1600536808032005/xu2456Isup2.hkl

Additional supplementary materials:  crystallographic information; 3D view; checkCIF report

## Figures and Tables

**Table 1:** Hydrogen-bond geometry (Å, °)

*D*—H⋯*A*	*D*—H	H⋯*A*	*D*⋯*A*	*D*—H⋯*A*
C6—H6⋯O4^i^	0.93	2.43	3.325 (4)	163
C8—H8⋯O2^ii^	0.93	2.48	3.293 (5)	145

Symmetry codes: (i) 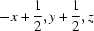; (ii) 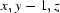.

**Fig. 1.**
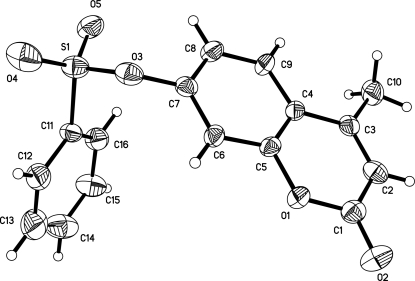
The molecular structure of the title compound. Displacement ellipsoids are drawn at the 30% probability level.

**Fig. 2.**
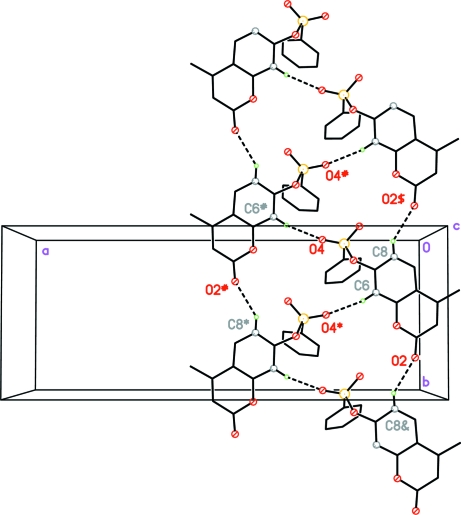
The crystal structure of the title compound, showing the formation of a hydrogen-bonded R33(18) ribbon along [010]. For clarity, H atoms not involving in H-bonding have been omitted. Dashed lines indicate hydrogen bonds [Symmetry codes: (*) 1/2 - x, 1/2 + y, z; (#) 1/2 - x, -1/2 + y, z; (&) x, 1 + y, x; ($) x, -1 + y, z].

## supplementary crystallographic information

### Comment

Coumarin derivatives exhibit a wide variety of pharmacological activities including anti-HIV (Xie *et al.*, 2001), antibacterial (Tanitame *et al.*, 2004), antioxidant (Shao *et al.*, 1997), antithrombotic (Rendenbach-Müller *et al.*, 1994) and antiinflammatory (Pochet *et al.*, 1996) activities. We have recently reported the crystal structures of some coumarin derivatives (Yang *et al.*, 2007, 2006). As part of our study of the crystal structures of coumarin derivatives, we report here the crystal structure of the title coumarin derivative.

The molecular structure is shown in Fig. 1. The dihedral angle between the coumarin ring system and the phenyl ring is 65.9 (1)°. The terminal S═O bond distances of 1.411 (3) and 1.421 (3) Å agree with 1.4207 (19) and 1.4331 (19) Å found in a related compound, 4-methyl-7-phenylsulfonamido-2*H*-1-benzopyran-2-one (Yang *et al.*, 2007).

In the crystal the molecules are linked by weak C—H···O hydrogen bonding to form the ribbon structure (Table 1 and  Fig. 2).

### Experimental

To an anhydrous pyridine solution (10 ml) of 7-hydroxy-4-methyl-coumarin (1.76 g, 10 mmol), a solution of phenylsulfonyl chloride (11 mmol) was slowly added at 278–283 K with stirring for 30 min. The reaction mixture was stirred continuously for 12 h at room temperature and then poured into ice–water (200 ml). The solid obtained was filtered off, washed with water and dried at room temperature. Colorless crystals of the title compound suitable for X-ray structure analysis were obtained by evaporation of an ethanol solution over a period of one week.

### Refinement

H atoms were placed in calculated positions with C—H = 0.93 Å (aromatic) and 0.96 Å (methyl), and refined in riding mode with *U*_iso_(H) = 1.2*U*_eq_(C) (aromatic) and *U*_iso_(H) = 1.5*U*_eq_(C) (methyl).

### Crystal data

**Table:**

C_16_H_12_O_5_S	*D*_x_ = 1.445 Mg m^−^^3^
*M**_r_* = 316.32	Melting point: 493 K
Orthorhombic, *P**b**c**n*	Mo *K*α radiation, λ = 0.71073 Å
Hall symbol: -P 2n 2ab	Cell parameters from 1900 reflections
*a* = 23.319 (3) Å	θ = 2.4–22.8°
*b* = 9.0865 (12) Å	µ = 0.24 mm^−^^1^
*c* = 13.7280 (17) Å	*T* = 298 K
*V* = 2908.8 (6) Å^3^	Block, colourless
*Z* = 8	0.48 × 0.35 × 0.23 mm
*F*(000) = 1312	

### Data collection

**Table:**

Siemens SMART CCD area-detector diffractometer	2557 independent reflections
Radiation source: fine-focus sealed tube	1340 reflections with *I* > 2σ(*I*)
graphite	*R*_int_ = 0.077
φ and ω scans	θ_max_ = 25.0°, θ_min_ = 1.8°
Absorption correction: multi-scan (SADABS; Sheldrick, 1996)	*h* = −27→25
*T*_min_ = 0.892, *T*_max_ = 0.946	*k* = −6→10
11238 measured reflections	*l* = −16→15

### Refinement

**Table:**

Refinement on *F*^2^	Primary atom site location: structure-invariant direct methods
Least-squares matrix: full	Secondary atom site location: difference Fourier map
*R*[*F*^2^ > 2σ(*F*^2^)] = 0.049	Hydrogen site location: inferred from neighbouring sites
*wR*(*F*^2^) = 0.164	H-atom parameters constrained
*S* = 1.09	*w* = 1/[σ^2^(*F*_o_^2^) + (0.0635*P*)^2^ + 1.4*P*] where *P* = (*F*_o_^2^ + 2*F*_c_^2^)/3
2557 reflections	(Δ/σ)_max_ < 0.001
200 parameters	Δρ_max_ = 0.21 e Å^−^^3^
0 restraints	Δρ_min_ = −0.27 e Å^−^^3^

### Special details

**Table:**

Geometry. All e.s.d.'s (except the e.s.d. in the dihedral angle between two l.s. planes) are estimated using the full covariance matrix. The cell e.s.d.'s are taken into account individually in the estimation of e.s.d.'s in distances, angles and torsion angles; correlations between e.s.d.'s in cell parameters are only used when they are defined by crystal symmetry. An approximate (isotropic) treatment of cell e.s.d.'s is used for estimating e.s.d.'s involving l.s. planes.
Refinement. Refinement of *F*^2^ against ALL reflections. The weighted *R*-factor *wR* and goodness of fit *S* are based on *F*^2^, conventional *R*-factors *R* are based on *F*, with *F* set to zero for negative *F*^2^. The threshold expression of *F*^2^ > σ(*F*^2^) is used only for calculating *R*-factors(gt) *etc*. and is not relevant to the choice of reflections for refinement. *R*-factors based on *F*^2^ are statistically about twice as large as those based on *F*, and *R*- factors based on ALL data will be even larger.

### Fractional atomic coordinates and isotropic or equivalent isotropic displacement parameters (Å^2^)

**Table:**

	*x*	*y*	*z*	*U*_iso_*/*U*_eq_	
O1	0.06623 (10)	0.5712 (3)	0.1179 (2)	0.0475 (7)	
O2	0.01987 (13)	0.7814 (3)	0.1253 (2)	0.0753 (10)	
O3	0.17597 (10)	0.1409 (3)	0.1092 (2)	0.0554 (8)	
O4	0.25437 (12)	0.0001 (4)	0.0562 (3)	0.0813 (11)	
O5	0.15694 (13)	−0.0921 (3)	0.0244 (2)	0.0686 (9)	
S1	0.19688 (4)	0.02586 (12)	0.02911 (9)	0.0554 (4)	
C1	0.01573 (17)	0.6497 (5)	0.1241 (3)	0.0511 (11)	
C2	−0.03663 (17)	0.5658 (5)	0.1266 (3)	0.0518 (11)	
H2	−0.0712	0.6168	0.1297	0.062*	
C3	−0.03837 (15)	0.4190 (4)	0.1249 (3)	0.0443 (10)	
C4	0.01530 (14)	0.3395 (4)	0.1195 (3)	0.0402 (9)	
C5	0.06587 (16)	0.4202 (4)	0.1156 (3)	0.0397 (9)	
C6	0.11874 (15)	0.3532 (4)	0.1088 (3)	0.0430 (10)	
H6	0.1521	0.4088	0.1047	0.052*	
C7	0.12071 (15)	0.2032 (4)	0.1081 (3)	0.0431 (10)	
C8	0.07195 (17)	0.1177 (4)	0.1123 (3)	0.0529 (11)	
H8	0.0743	0.0155	0.1116	0.063*	
C9	0.01984 (17)	0.1871 (4)	0.1176 (3)	0.0514 (11)	
H9	−0.0134	0.1306	0.1199	0.062*	
C10	−0.09430 (15)	0.3386 (5)	0.1251 (3)	0.0669 (13)	
H10A	−0.1251	0.4079	0.1317	0.100*	
H10B	−0.0951	0.2708	0.1787	0.100*	
H10C	−0.0985	0.2855	0.0651	0.100*	
C11	0.19416 (16)	0.1231 (4)	−0.0797 (3)	0.0444 (10)	
C12	0.24038 (18)	0.2080 (5)	−0.1072 (4)	0.0699 (14)	
H12	0.2721	0.2163	−0.0665	0.084*	
C13	0.2393 (3)	0.2794 (6)	−0.1940 (5)	0.0937 (18)	
H13	0.2705	0.3363	−0.2131	0.112*	
C14	0.1923 (3)	0.2683 (6)	−0.2538 (4)	0.0906 (18)	
H14	0.1919	0.3175	−0.3132	0.109*	
C15	0.1467 (2)	0.1859 (6)	−0.2267 (4)	0.0723 (14)	
H15	0.1149	0.1795	−0.2675	0.087*	
C16	0.14680 (18)	0.1116 (5)	−0.1394 (3)	0.0583 (12)	
H16	0.1155	0.0547	−0.1209	0.070*	

### Atomic displacement parameters (Å^2^)

**Table:**

	*U*^11^	*U*^22^	*U*^33^	*U*^12^	*U*^13^	*U*^23^
O1	0.0504 (16)	0.0403 (16)	0.0519 (19)	−0.0075 (12)	−0.0003 (14)	−0.0038 (13)
O2	0.089 (2)	0.0449 (19)	0.093 (3)	0.0008 (16)	−0.0127 (19)	−0.0033 (18)
O3	0.0567 (16)	0.0623 (18)	0.0473 (19)	0.0100 (14)	−0.0067 (14)	−0.0124 (15)
O4	0.0632 (18)	0.099 (3)	0.082 (2)	0.0411 (17)	−0.0239 (17)	−0.0130 (19)
O5	0.084 (2)	0.0457 (17)	0.076 (2)	−0.0071 (15)	0.0033 (18)	−0.0047 (16)
S1	0.0583 (7)	0.0514 (6)	0.0566 (8)	0.0129 (5)	−0.0072 (6)	−0.0057 (6)
C1	0.063 (3)	0.050 (3)	0.041 (3)	−0.003 (2)	−0.005 (2)	−0.003 (2)
C2	0.050 (2)	0.067 (3)	0.039 (3)	0.007 (2)	−0.003 (2)	−0.007 (2)
C3	0.045 (2)	0.056 (3)	0.032 (2)	−0.0075 (19)	0.0005 (18)	−0.008 (2)
C4	0.045 (2)	0.045 (2)	0.030 (2)	−0.0130 (18)	0.0001 (18)	−0.0077 (18)
C5	0.050 (2)	0.037 (2)	0.031 (2)	−0.0074 (18)	−0.0024 (19)	−0.0035 (17)
C6	0.045 (2)	0.045 (2)	0.039 (2)	−0.0116 (18)	0.0002 (18)	−0.0040 (19)
C7	0.050 (2)	0.045 (2)	0.034 (2)	0.0005 (19)	0.0018 (19)	−0.0047 (19)
C8	0.065 (3)	0.037 (2)	0.057 (3)	−0.007 (2)	0.003 (2)	−0.003 (2)
C9	0.053 (3)	0.044 (2)	0.057 (3)	−0.018 (2)	0.003 (2)	−0.006 (2)
C10	0.047 (2)	0.083 (3)	0.071 (4)	−0.016 (2)	0.003 (2)	−0.011 (3)
C11	0.043 (2)	0.043 (2)	0.047 (3)	0.0069 (19)	−0.001 (2)	−0.009 (2)
C12	0.061 (3)	0.083 (3)	0.066 (4)	−0.014 (3)	0.003 (3)	−0.018 (3)
C13	0.113 (5)	0.099 (4)	0.069 (4)	−0.029 (4)	0.031 (4)	−0.004 (4)
C14	0.146 (6)	0.071 (4)	0.055 (4)	0.016 (4)	0.016 (4)	0.002 (3)
C15	0.090 (4)	0.073 (3)	0.055 (4)	0.022 (3)	−0.015 (3)	−0.007 (3)
C16	0.054 (3)	0.058 (3)	0.063 (3)	0.003 (2)	−0.004 (2)	−0.005 (2)

### Geometric parameters (Å, °)

**Table:**

O1—C5	1.373 (4)	C7—C8	1.378 (5)
O1—C1	1.379 (4)	C8—C9	1.371 (5)
O2—C1	1.201 (4)	C8—H8	0.9300
O3—C7	1.407 (4)	C9—H9	0.9300
O3—S1	1.594 (3)	C10—H10A	0.9600
O4—S1	1.411 (3)	C10—H10B	0.9600
O5—S1	1.421 (3)	C10—H10C	0.9600
S1—C11	1.736 (4)	C11—C12	1.378 (5)
C1—C2	1.440 (5)	C11—C16	1.379 (5)
C2—C3	1.334 (5)	C12—C13	1.358 (7)
C2—H2	0.9300	C12—H12	0.9300
C3—C4	1.447 (5)	C13—C14	1.372 (7)
C3—C10	1.495 (5)	C13—H13	0.9300
C4—C9	1.389 (5)	C14—C15	1.354 (7)
C4—C5	1.390 (4)	C14—H14	0.9300
C5—C6	1.378 (5)	C15—C16	1.376 (6)
C6—C7	1.364 (5)	C15—H15	0.9300
C6—H6	0.9300	C16—H16	0.9300
			
C5—O1—C1	120.9 (3)	C9—C8—H8	120.8
C7—O3—S1	122.4 (2)	C7—C8—H8	120.8
O4—S1—O5	120.6 (2)	C8—C9—C4	121.8 (3)
O4—S1—O3	102.59 (17)	C8—C9—H9	119.1
O5—S1—O3	109.02 (17)	C4—C9—H9	119.1
O4—S1—C11	110.2 (2)	C3—C10—H10A	109.5
O5—S1—C11	108.70 (19)	C3—C10—H10B	109.5
O3—S1—C11	104.37 (16)	H10A—C10—H10B	109.5
O2—C1—O1	116.6 (4)	C3—C10—H10C	109.5
O2—C1—C2	126.5 (4)	H10A—C10—H10C	109.5
O1—C1—C2	116.9 (3)	H10B—C10—H10C	109.5
C3—C2—C1	123.7 (4)	C12—C11—C16	120.4 (4)
C3—C2—H2	118.2	C12—C11—S1	119.5 (3)
C1—C2—H2	118.2	C16—C11—S1	120.1 (3)
C2—C3—C4	118.3 (3)	C13—C12—C11	119.5 (5)
C2—C3—C10	121.0 (4)	C13—C12—H12	120.2
C4—C3—C10	120.7 (4)	C11—C12—H12	120.2
C9—C4—C5	117.4 (3)	C12—C13—C14	120.4 (5)
C9—C4—C3	124.4 (3)	C12—C13—H13	119.8
C5—C4—C3	118.2 (3)	C14—C13—H13	119.8
O1—C5—C6	115.9 (3)	C15—C14—C13	120.3 (5)
O1—C5—C4	122.1 (3)	C15—C14—H14	119.9
C6—C5—C4	121.9 (3)	C13—C14—H14	119.9
C7—C6—C5	118.2 (3)	C14—C15—C16	120.5 (5)
C7—C6—H6	120.9	C14—C15—H15	119.7
C5—C6—H6	120.9	C16—C15—H15	119.7
C6—C7—C8	122.4 (3)	C15—C16—C11	118.9 (4)
C6—C7—O3	115.6 (3)	C15—C16—H16	120.6
C8—C7—O3	121.9 (3)	C11—C16—H16	120.6
C9—C8—C7	118.3 (4)		
			
C7—O3—S1—O4	177.3 (3)	C5—C6—C7—O3	−174.9 (3)
C7—O3—S1—O5	−53.7 (3)	S1—O3—C7—C6	−125.2 (3)
C7—O3—S1—C11	62.3 (3)	S1—O3—C7—C8	58.8 (5)
C5—O1—C1—O2	−179.7 (4)	C6—C7—C8—C9	−0.1 (6)
C5—O1—C1—C2	−0.8 (5)	O3—C7—C8—C9	175.6 (4)
O2—C1—C2—C3	179.8 (4)	C7—C8—C9—C4	−0.6 (6)
O1—C1—C2—C3	1.1 (6)	C5—C4—C9—C8	0.2 (6)
C1—C2—C3—C4	−0.4 (6)	C3—C4—C9—C8	179.9 (4)
C1—C2—C3—C10	−178.4 (4)	O4—S1—C11—C12	−23.7 (4)
C2—C3—C4—C9	179.7 (4)	O5—S1—C11—C12	−157.9 (3)
C10—C3—C4—C9	−2.2 (6)	O3—S1—C11—C12	85.8 (3)
C2—C3—C4—C5	−0.6 (5)	O4—S1—C11—C16	154.3 (3)
C10—C3—C4—C5	177.5 (4)	O5—S1—C11—C16	20.0 (4)
C1—O1—C5—C6	179.6 (3)	O3—S1—C11—C16	−96.2 (3)
C1—O1—C5—C4	−0.1 (5)	C16—C11—C12—C13	−0.6 (6)
C9—C4—C5—O1	−179.5 (3)	S1—C11—C12—C13	177.4 (4)
C3—C4—C5—O1	0.8 (5)	C11—C12—C13—C14	0.5 (8)
C9—C4—C5—C6	0.8 (6)	C12—C13—C14—C15	0.0 (8)
C3—C4—C5—C6	−178.9 (3)	C13—C14—C15—C16	−0.4 (8)
O1—C5—C6—C7	178.8 (3)	C14—C15—C16—C11	0.3 (7)
C4—C5—C6—C7	−1.5 (6)	C12—C11—C16—C15	0.2 (6)
C5—C6—C7—C8	1.1 (6)	S1—C11—C16—C15	−177.8 (3)

### Hydrogen-bond geometry (Å, °)

**Table:**

*D*—H···*A*	*D*—H	H···*A*	*D*···*A*	*D*—H···*A*
C6—H6···O4^i^	0.93	2.43	3.325 (4)	163
C8—H8···O2^ii^	0.93	2.48	3.293 (5)	145

Symmetry codes: (i) −*x*+1/2, *y*+1/2, *z*; (ii) *x*, *y*−1, *z*.
